# Brain Viscoelasticity Alteration in Chronic-Progressive Multiple Sclerosis

**DOI:** 10.1371/journal.pone.0029888

**Published:** 2012-01-20

**Authors:** Kaspar-Josche Streitberger, Ingolf Sack, Dagmar Krefting, Caspar Pfüller, Jürgen Braun, Friedemann Paul, Jens Wuerfel

**Affiliations:** 1 Department of Radiology, Charité – University Medicine Berlin, Berlin, Germany; 2 NeuroCure Clinical Research Center and Experimental and Clinical Research Center, Charité – University Medicine Berlin and Max Delbrueck Center for Molecular Medicine, Berlin, Germany; 3 Institute of Medical Informatics, Charité – University Medicine Berlin, Berlin, Germany; 4 Institute of Neuroradiology, University Luebeck, Luebeck, Germany; 5 Clinical and Experimental Multiple Sclerosis Research Center, Charité - University Medicine Berlin, Berlin, Germany; University of Maryland, United States of America

## Abstract

**Introduction:**

Viscoelastic properties indicate structural alterations in biological tissues at multiple scales with high sensitivity. Magnetic Resonance Elastography (MRE) is a novel technique that directly visualizes and quantitatively measures biomechanical tissue properties *in vivo*. MRE recently revealed that early relapsing-remitting multiple sclerosis (MS) is associated with a global decrease of the cerebral mechanical integrity. This study addresses MRE and MR volumetry in chronic-progressive disease courses of MS.

**Methods:**

We determined viscoelastic parameters of the brain parenchyma in 23 MS patients with primary or secondary chronic progressive disease course in comparison to 38 age- and gender-matched healthy individuals by multifrequency MRE, and correlated the results with clinical data, T2 lesion load and brain volume. Two viscoelastic parameters, the shear elasticity *μ* and the powerlaw exponent *α*, were deduced according to the springpot model and compared to literature values of relapsing-remitting MS.

**Results:**

In chronic-progressive MS patients, *μ* and *α* were reduced by 20.5% and 6.1%, respectively, compared to healthy controls. MR volumetry yielded a weaker correlation: Total brain volume loss in MS patients was in the range of 7.5% and 1.7% considering the brain parenchymal fraction. All findings were significant (P<0.001).

**Conclusions:**

Chronic-progressive MS disease courses show a pronounced reduction of the cerebral shear elasticity compared to early relapsing-remitting disease. The powerlaw exponent *α* decreased only in the chronic-progressive stage of MS, suggesting an alteration in the geometry of the cerebral mechanical network due to chronic neuroinflammation.

## Introduction

Magnetic resonance imaging (MRI) has emerged as most important paraclinical tool for the diagnosis and monitoring of disease activity in multiple sclerosis (MS), which is reflected by the current MS diagnostic criteria [1], [2]. However, disease specificity of conventional MRI parameters, such as T2 lesion load, is limited [3] and their association with clinical course and neurological disability is only modest [4]. Consequently, considerable scientific effort is necessary to further improve the diagnostic specificity and predictive value of MRI in MS.

In a recent development of medical imaging, the sensitivity of viscoelastic constants to the hierarchy of the mechanical matrix of tissue is exploited. Mechanical imaging, also called elastography [5], promises sensitivity to pathological processes which affect the tissue architecture across a continuum of microscopical to macroscopical scales. To date, only MR elastography (MRE) [6] is capable of measuring the viscoelastic properties of living brain in its natural environment [7], [8], [9]. In cerebral MRE, harmonic vibrations are applied to the skull; the induced shear waves inside the brain are subsequently captured by motion sensitive MRI sequences. Thus, the obtained frequency-resolved complex wave images can be analyzed by wave inversion for viscoelastic parameter recovery. Multifrequency MRE combined with two-parameter springpot analyses have recently been applied in brain studies of aging [10], [11], normal pressure hydrocephalus [12], [13] and MS [14]. The reported springpot parameters *μ* and *α* describe the viscoelastic dispersion of the complex shear modulus G* of brain tissue by a powerlaw, i.e. a linear function in logarithmical coordinates. The observed linear increase of log(G*) over log(*ω*),with *ω* being the angular drive frequency, implies scaling of G* and therewith a hierarchical order of the mechanical network in brain tissue [10]. The shear elasticity *μ* is related to the inherent strength or integrity of this network, while the powerlaw exponent *α* is related to geometry, i.e. the topology or fractal dimension of the network [15], [16]. Both springpot parameters are potentially sensitive markers of a variety of neurological disorders affecting the global mechanical scaffold of brain at multiple scales.

Our primary hypothesis is based on a recently published study of multifrequency MRE applied to a group of patients suffering from relapsing-remitting MS [14]. At this early state of MS, a significant decrease of *μ* compared to healthy controls was observed while *α* remained unchanged. In a preliminary interpretation of these results, it was speculated that the network integrity of the brain is affected due to widespread neurodegenerative processes in MS, whereas the geometry of this network is not. We therefore hypothesize that chronic inflammation in progressive MS causes a further progression of brain matrix degradation, resulting in a reduction of both, *μ* and *α*. Our secondary hypothesis is that the parameters *μ* and *α* may reflect neurodegenerative processes in MS due to the sensitivity of biomechanical parameters to structural changes also on the microscopic level. Thus, MRE parameters may yield a higher sensitivity to neurodegenerative processes in comparison to, e.g. MR volumetry [17].

To test both hypotheses, patients with chronic-progressive MS disease course and matched healthy volunteers were enrolled in a cross-sectional study, combining multifrequency MRE and MR volumetry. Supplementary data were acquired by standard clinical and neuroradiological methods. We compared our resulting data to literature values in relapsing-remitting forms of MS. Thus, conclusions could be drawn about the impact of early relapsing-remitting and chronic-progressive MS disease courses on brain mechanical constants.

## Methods

### Study participants

Twenty-three consecutive patients with primary (n = 6), or secondary (n = 17) chronic progressive disease course were recruited for this study from the NeuroCure Clinical Research Center or the neuroimmunology outpatient clinic of the Experimental and Clinical Research Center. Demographic data are summarized in Table 1. The study was approved by the ethics committee of the Charité – University Medicine Berlin, and all participants gave written informed consent. A cohort of thirty-eight age and gender matched healthy volunteers without neurological or psychiatric diseases served as controls.

**Table 1:** Demographical data, clinical characteristics, brain volumes, brain parenchymal fraction (BPF) and viscoelastic constants *μ* and *α* according to the springpot model.

group	MS (sp)/MS (pp)	control	MS (rr)*	control*
N	17 (sp)/6 (pp)	38	45	34
female N	9 (sp)/4 (pp)	22	23	17
age	52 (9.1)/51 (5.0)	48 (9.7)	38 (8.0)	37 (11.4)
Mean EDSS	5.6 (1.3)/5.3 (1.8)	0	1.6 (1.4)	0
V in dm^3^	1.513 (0.178)	1.636 (0.068)	n.a.	n.a.
ΔV	−0.123			
	−7.52%			
	R = −0.4462			
	P<0.001			
*μ* (in kPa)	2.607 (0.482)	3.278 (0.314)	3.025 (0.459)	3.545 (0.556)
Δ*μ*	−0.67		−0.52	
	−20.46%		−14.68%	
	R = −0.6515		R = −0.4604	
	P<0.001		P<0.001	
*α*	0.2756 (0.0108)	0.2934 (0.0086)	0.2937 (0.0129)	0.2928 (0.0131)
Δ*α*	−0.0178		0.0009	
	−6.07%		0.29%	
	R = −0.6786		R = 0.0333	
	P<0.001		P = 0.770	

MS – multiple sclerosis; sp – secondary progressive; pp – primary progressive; rr – relapsing remitting; EDSS – expanded disability status scale; n.a. – not applicable; standard deviations are given in brackets;

*data taken from [14] and processed corresponding to the data of progressive MS.

### Magnetic resonance elastography

**Figure 1:**
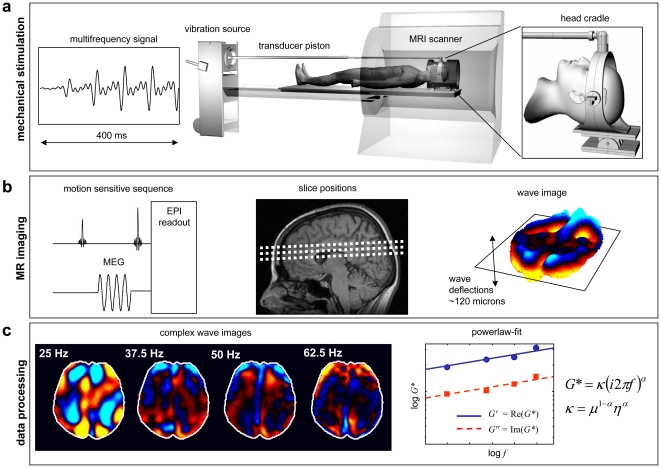
Scheme of cerebral multifrequency MRE. **a:** The MRI scanner is combined with a device for acoustical head stimulations comprising: 1) a signal generator that produces a multifrequency signal composed from four harmonic frequencies of 25, 37.5, 50 and 62.5 Hz; 2) a loudspeaker for generating acoustic vibrations; 3) an extended piston that transfers the vibrations into the scanner and 4) a head cradle for stimulating head vibrations mainly along the head-feet direction. **b:** A single-shot echo planar imaging (EPI) sequence is sensitized to harmonic motions by a 60-Hz sinusoidal motion encoding gradient (MEG) of four cycles and directed through-plane. The image planes are positioned in transverse orientation through the brain (parallel to the “anterior and posterior commissure line (AC-PC)”) in a central slab of the brain. The resulting wave images display the motion component along the head-feet direction corresponding to the major vibration direction of the actuator. **c:** Image processing comprises Fourier decomposition of the superposed oscillations yielding four complex single-frequency wave images, corresponding to the experimentally applied vibration frequencies. Each of the wave images is separately inverted, resulting in four complex-valued shear modulus images, whose values are averaged within a region of interest comprising the parenchyma within the image slice (demarcated in the wave images by white lines).

MRI measurements were performed on a 1.5 tesla scanner (Sonata, Siemens Medical Systems, Erlangen, Germany). A detailed scheme of the experiment is presented in Figure 1. The MRE protocol comprised a single-shot spin-echo echo planar imaging sequence with a sinusoidal motion-encoding gradient (MEG) in through-plane direction (*z*-direction) that was used to acquire three transversal image slices in a central slab through the cerebrum (number of MEG cycles: 4, MEG amplitude: 35 mT/m, repetition time [TR]: 3.0 s, echo time [TE]: 149 ms, field of view [FoV]: 192×192 mm^2^, matrix size: 128×128, slice thickness: 6 mm).

For each slice, the acquisition was repeated 64 times during one period of 80 ms. A toggle gradient with alternating sign of motion sensitization was applied with a stepwise increasing delay between vibration onset and motion-encoding of 2.5 ms. A multifrequency vibration with maximum amplitude of approximately 0.5 mm in parallel direction to the long axis of the magnet was fed into an actuator by a carbon fiber piston. The resulting time-resolved wave images, *u*(*x*,*y*,*t*) (with *x* and *y* as spatial coordinates) were Fourier-transformed for decomposition into complex wave images at drive frequency: *U*(*x*,*y*,*ω*); *ω* = 2*πf* with *f* being the drive frequency. *U*(*x*,*y*,*ω*) was subjected to spatial filtering to suppress compression wave components and noise using isotropic lower (upper) thresholds of 5.56 (50.0) m^−1^, 8.33 (66.67) m^−1^, 10.0 (90.9) m^−1^, and 10.0 (100.0) m^−1^ for *f* = 25 Hz, 37.5 Hz, 50 Hz and 62.5 Hz, respectively. These bandpass filters were applied to the masked wave images excluding air outside the brain but including CSF and ventricles. Complex modulus images were obtained by wave inversion (G*(*x*,*y*,*ω*) = −*ρω* ^2^ *U*/Δ*U*), with Δ as the 2D-Laplace operator and *ρ* being the tissue's density of 1 kg/dm^3^), spatially averaged within the segmented brain parenchyma. The resulting global modulus function was fitted by a least-square routine. A good match between model and multifrequency data was reached by a combination of Voigt and Maxwell models given by the three-parameter Zener model. However, the latter model incorporated an additional parameter – a second shear modulus – rendering the interpretation of viscoelastic constants rather cumbersome. The optimal trade-off between physical significance and representation of the frequency dependency of our data was achieved by a two-parameter springpot model G* = *κ*(*iω*)^α^, that represents a powerlaw and interpolates between springs and dashpots introducing a fractional element *κ* = *μ* ^(1−*α*)^ *η^α^* [10]. Two springpot variables are represented by *μ* and *α*, while *η* is the viscosity of the medium, which has to be defined *a priori* in order to derive shear moduli comparable to other brain mechanical tests. In [10] it was suggested to use *η* = 3.7 Pa·s for human brain, which is the viscosity value of the brain parenchyma derived by the Zener model, and was also used in this study. Every participant underwent MRE of approximately 15 minutes duration additionally to a routine clinical MRI protocol: T2- and proton density-weighted images (TR: 5780 ms, TE_1_: 13 ms, TE_2_: 81 ms, TE_3_: 121 ms; 3 mm slice thickness, no gap, 44 contiguous axial slices). Bulk white matter lesion load of T2-weighted scans were routinely measured using the MedX v.3.4.3 software package, as described previously [18].

### Assessment of brain volume and calculation of the brain parenchymal fraction

Total brain tissue volumes, normalised for subject head size, were estimated on three-dimensional T1-weighted sequences (TR: 2110 ms, TE: 4.38 ms, inversion time [TI] 1100 ms, flip angle 15°, resolution 1 mm^3^), applying SIENAX [19], [20], [21], part of FSL [22]. SIENAX extracted brain and skull images from single whole-head input data [19]. The brain image was then affine-registered to MNI152 space [23], [24], using the skull image to determine the registration scaling. This was primarily in order to obtain the volumetric scaling factor to be used as normalization for head size. Subsequently, tissue-type segmentation with partial volume estimation was carried out [25] to calculate the total volume of the brain tissue, including separate estimates of volumes of grey matter, white matter, peripheral gray matter and ventricular CSF. Brain parenchymal fraction (BPF) is expressed as ratio of the sum of white and grey brain matter by intracranial volume.

### Statistical analysis

All data were analyzed using IBM SPSS Statistics 18 (IBM Corporation, Route 100, Somers, NY, USA). Correlation between clinical parameters, MRI and MRE was assessed by Spearman's correlation coefficient. Differences between patients and controls regarding age, MRI and MRE parameters were analyzed by the Mann-Whitney U test. Multivariate linear regression analyses were performed to assess the influence of MRI and MRE and clinical parameters on the springpot parameter *μ*. Owing to the study design, all tests should be understood as constituting exploratory data analysis, such that no adjustments for multiple testing were made. Receiver operating curve (ROC) analyses were performed in Matlab (MATLAB 6.5 R13, The MathWorks, Natick, MA, USA). Preliminary cut-off values for *μ* and *α* were attained by the maximum Youden index.

## Results

### Reduced brain volumes in MS patients compared to healthy controls

In accordance to the literature [26], MS patients exhibited significantly lower brain parenchymal volumes expressed as compared to healthy controls, corresponding to an average reduction of 7.5% (P<0.001) in patients.

BPF in MS patients (0.9610) was 1.69% lower than in healthy controls (0.9775; P<0.001). As these relative values depend on the grey matter/white matter contrast, we were not able to reliably compare our present data to a previous study on relapsing-remitting MS patients that was performed on a different MR scanner system.

**Figure 2:**
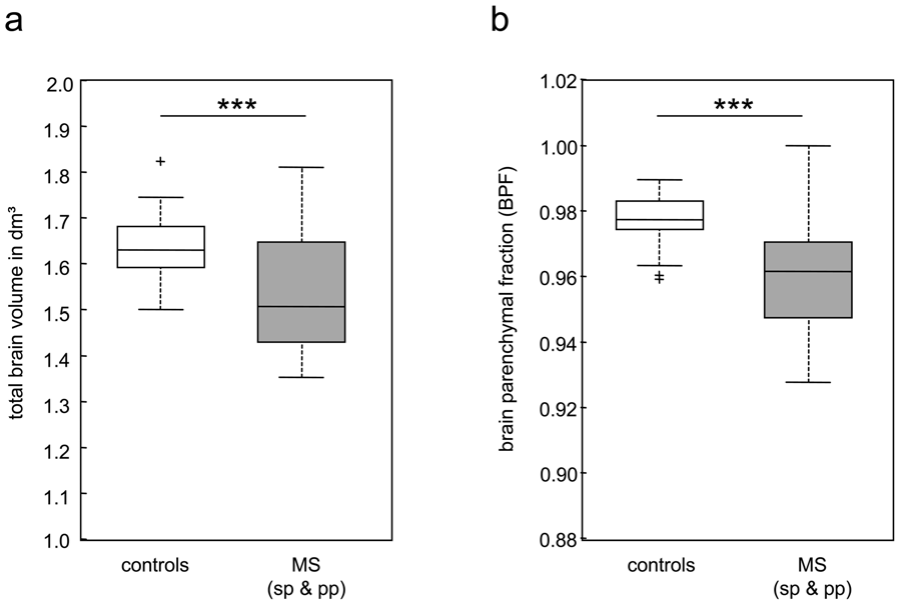
Brain atrophy in MS patients. Significantly reduced brain parenchymal volume (**a**) and brain parenchymal fraction (BPF) (**b**) in MS patients compared to matched healthy individuals (*** P<0.001). The boxplots depict the lower and upper quartiles as well as the 50^th^ percentile (median). Full data range is presented by the whiskers. sp – secondary progressive, pp – primary progressive, rr – relapsing remitting.

The volume- and BPF data are plotted in figure 2. Group values are given in table 1.

### Reduction of viscoelasticity constants in MS patients

**Figure 3:**
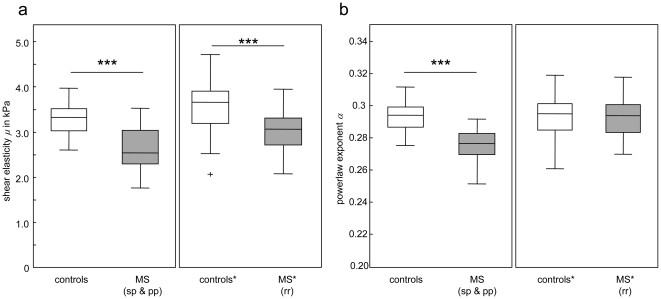
Reduction of brain parenchymal viscoelastic constants. MS patients present with significantly reduced brain parenchymal elasticity *μ* (**a**, P<0.001), but also with a reduction in the powerlaw exponent *α* (**b**, P<0.001) in MS patients with progressive disease course. The boxplot depicts the lower and upper quartiles as well as the 50^th^ percentile (median). Full data range is presented by the whiskers. sp – secondary progressive, pp – primary progressive, rr – relapsing remitting; *data for rr-MS are taken from [14] and reprocessed according to the methods reported in herein.

Both springpot constants *μ and α* were significantly lower in MS patients compared to healthy controls, corresponding to average reductions of 20.46% and 6.07%, respectively (P always<0.001). The group response is plotted in figure 3. Averaged values of *μ* and *α* as well as their disease related alterations are tabulated.

### Correlation of viscoelasticity constants with age and brain volume

In the MS group, the shear elasticity *μ* did neither correlate with age (R = 0.133, P = 0.545), nor with the BPF (R = 0.220, P = 0.313). Also no correlation was seen between *μ* and the total brain volume (R = 0.375, P = 0.077). Also *α* was not correlated to age (R = −0.210, P = 0.335), BPF (R = 0.017, P = 0.936) and volume (R = 0.021, P = 0.924). In this group, no correlation was seen between age and brain volume (R = 0.323, P = 0.133) and age and BPF (R = −0.118, P = 0.593). In the group of healthy volunteers, *μ* correlated with age (R = −0.607, P<0.001) and volume (R = 0.481, P = 0.002) while between *μ* and BPF only a trend was discernable (R = 0.309, P = 0.059). In agreement to MRE studies in the literature, *α* does not change with age in healthy volunteers (R = −0.124, P = 0.459) [10], [11], neither it does with volume (R = 0.104, P = 0.535) nor BPF (R = 0.117, P = 0.482). Within the patient group, there was no correlation between number or volume of whole brain T2 lesions with any of the viscoelasticity parameters (data not shown). However, five datasets needed to be removed from the semi-automated T2 analysis due to movement artefacts, resulting in a small number of datasets, warranting a repetition in a larger trial.

In a multivariate linear regression analysis including age, BPF and total brain volume as covariates, none of these variables independently predicted elasticity parameter *μ* in the MS group (standardized coefficients Beta = 0.044, P = 0.845; Beta = 0.214, P = 0.320; Beta = 0.355, P = 0.122, respectively).

Combining both groups, *μ* is correlated with age (R = −0.337, P = 0.008), brain volume (R = 0.559, P<0.001) and BPF (R = 0.502, P<0.001). *α* displays only a trend with age (R = −0.249, P = 0.053) but is correlated to brain volume (R = 0.333, P = 0.009) and BPF (R = 0.394, P = 0.002).

### Correlation of MRE and MRI parameters with clinical data

Not surprisingly, EDSS (expanded disability status scale) correlated with disease duration (R = 0.506, P = 0.014), but not with BPF and total brain volume (R = 0.02, P = 0.928; R = 0.05, P = 0.822, respectively). BPF inversely correlated with disease duration (R = −0.418, P = 0.047).

*μ* was neither correlated with EDSS (R = −0.077, P = 0.728) nor with disease duration (R = −0.190, P = 0.384). When including EDSS and disease duration as additional covariates in the multivariate linear regression analysis, none of these were independent predictors of *μ*. Similarly, *α* was neither correlated with EDSS (R = 0.099, P = 0.653) nor with disease duration (R = −0.381, P = 0.072).

### MRE differentiates healthy from diseased brains

**Figure 4:**
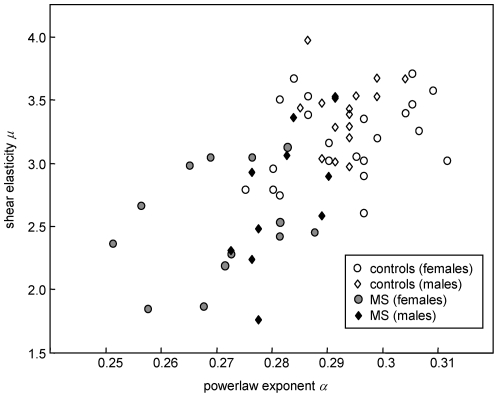
Viscoelastic constants for the detection of brain pathology. Individual data of shear elasticity *μ* and powerlaw exponent *α* of brain tissue in healthy volunteers and MS patients. The areas under the receiver characteristics curve (AUROC) for separating healthy volunteers from MS patients are 0.896 and 0.936 for *μ* and *α*, respectively.

In an area under the receiver operating characteristics curve analysis (AUROC), we found a high value for both the shear elasticity *μ* (0.896) and the powerlaw exponent *α* (0.936) for the discrimination of MS patients versus controls (Fig. 4). Preliminary cut-off values for diagnostic MRE in MS are proposed with *μ* = 2.677 kPa and *α* = 0.285. The corresponding sensitivity and specificity values are 0.896 and 0.658 and 0.936 and 0.842 for *μ* and *α*, respectively.

## Discussion

We investigated brain viscoelastic properties in patients with progressive disease courses of MS, and compared these to a group of healthy individuals of matched age and gender. The most important findings of our work are that (i) both, the shear elasticity *μ* and the powerlaw exponent *α* differentiate MS from healthy controls and (ii), although there is an association between brain atrophy and changes in viscoelasticity in the total study cohort, brain volume reduction is not the only contributor to the alterations of viscolelastic properties in MS. It has recently been demonstrated in a large group of healthy volunteers that brain atrophy only weakly contributes to viscoelastic constants measured by MRE [11]. The correlation between viscoelastic constants and atrophy found in our study is not surprising, since parenchymal degradation - physiologically during aging or pathologically accelerated in MS - impacts both, the mechanical matrix of brain and its volume. However, correlation does not necessarily imply causality. The fact that viscoelastic constants measured by MRE can represent volume independent markers is indicated by a higher rate of parameter changes in MRE compared to MR volumetry. However, at this state of research we cannot definitely exclude interactions between volumetrical changes, brain geometry and MRE. As a consequence, we have been focusing on averaged viscoelastic constants related to the whole parenchyma visible in the slab of the chosen image slices. As such, cerebral MRE still represents a global method providing data for widespread effects occult to standard imaging modalities. In MS, the measured springpot constants suggest that large regions of the central cerebrum (i.e. the region of our wave image slices) are mechanically degraded due to neuroinflammation. The reduction of the shear elasticity reported in [14] for early MS is enhanced in chronic-progressive MS (−12.7% versus −20.5%, see table 1). Most interestingly, *α* - which was not significantly correlated in early relapsing-remitting MS - is altered in chronic-progressive disease courses (0.29% versus 6.1%). This draws our attention to the structure sensitivity of *μ* and *α*. The principal relationship between powerlaw exponent *α* and the hierarchical architecture of biomechanical networks was illustrated in [16] by numerical simulations and multifrequency MRE experiments of skeletal muscle. It was stated there that an increase in the fractal dimension of the network (given in contracting muscle by the establishment of myosin cross bridges) yields to an increase of the powerlaw exponent *α* while *μ* is influenced by the network-inherent spring constants quantifying the mechanical integrity of the underlying tissue. Translating these findings to brain viscoelasticity in MS leads us to the following hypothesis: In early disease stages the integrity of the mechanical matrix is degraded while its inherent geometrical order is preserved. However, this order becomes affected during further disease progression, resulting in a continuous decrease of *μ*, but furthermore also to a reduction in *α*. The latter process may occur either in a slow and continuous manner, or by discontinuous remodelling of the tissue similar to a phase transition from an ordered to a disordered state of the brain parenchyma. To date it is not entirely clear which kind of mechanical cerebral tissue structure determines *μ* or *α*. Recent *in vivo* MRE experiments in mouse models suggested that both demyelination and inflammation contribute to the observed deterioration of the cerebral mechanical scaffold [27], [28].

Our study is limited by a relatively small number of patients, preventing an individual group analysis of secondary progressive and primary progressive MS. A further limitation lies in different protocols used between previous studies published in [10], [14], [29], and our current set-up [11], [12], [13]. We therefore reprocessed the data of [14] using the filter limits and the masking of wave images as given in the Methods section. Furthermore, the alignment of image slices in a peripheral slab of the brain through the upper part or slightly above the ventricles (as done in [10], [14], [29]) may result in a larger variation of viscoelastic constants than observed in a more central position as an effect of enhanced wave scattering. Our conclusive statements regarding early relapsing-remitting and chronic-progressive MS are therefore limited to relative effects between healthy controls and patients groups. Applying three-dimensional multifrequency MRE in future studies will alleviate current restrictions to the slice alignment and may therewith help to establish generalized viscoelastic thresholds for diagnostic applications of MRE.

In summary, we demonstrate that cerebral viscoelastic constants are reduced in chronic-progressive MS. In contrast to early relapsing-remitting MS disease courses, the springpot powerlaw exponent *α* is reduced in chronic-progressive MS, indicating a loss of the mechanical network geometry due to chronic neuroinflammation. MRI volumetry is less sensitive to changes in chronic-progressive MS and represents therewith only a minor predictor for viscoelastic constants measured by MRE.
